# Impact of neonatal resuscitation trainings on neonatal and perinatal mortality: a systematic review and meta-analysis

**DOI:** 10.1136/bmjpo-2017-000183

**Published:** 2017-11-16

**Authors:** Archana Patel, Mahalaqua Nazli Khatib, Kunal Kurhe, Savita Bhargava, Akash Bang

**Affiliations:** 1 Lata Medical Research Foundation, Nagpur, Maharashtra, India; 2 Division of Evidence Synthesis; School of Epidemiology and Public Health & Department of Physiology, Datta Meghe Institute of Medical Sciences, Wardha, Maharashtra, India; 3 Department of Paediatrics, Mahatma Gandhi Institute of Medical Sciences, Sewagram, Maharashtra, India

**Keywords:** health services research, mortality, multidisciplinary team-care

## Abstract

**Background:**

Training of birth attendants in neonatal resuscitation is likely to reduce birth asphyxia and neonatal mortality. We performed a systematic review and meta-analysis to assess the impact of neonatal resuscitation training (NRT) programme in reducing stillbirths, neonatal mortality, and perinatal mortality

**Methods:**

We considered studies where any NRT was provided to healthcare personnel involved in delivery process and handling of newborns. We searched MEDLINE, CENTRAL, ERIC and other electronic databases. We also searched ongoing trials and bibliographies of the retrieved articles, and contacted experts for unpublished work. We undertook screening of studies and assessment of risk of bias in duplicates. We performed review according to Cochrane Handbook. We assessed the quality of evidence using the GRADE approach.

**Results:**

We included 20 trials with 1 653 805 births in this meta-analysis. The meta-analysis of NRT versus control shows that NRT decreases the risk of all stillbirths by 21% (RR 0.79, 95% CI 0.44 to 1.41), 7-day neonatal mortality by 47% (RR 0.53, 95% CI 0.38 to 0.73), 28-day neonatal mortality by 50% (RR 0.50, 95% CI 0.37 to 0.68) and perinatal mortality by 37% (RR 0.63, 95% CI 0.42 to 0.94). The meta-analysis of pre-NRT versus post-NRT showed that post-NRT decreased the risk of all stillbirths by 12% (RR 0.88, 95% CI 0.83 to 0.94), fresh stillbirths by 26% (RR 0.74, 95% CI 0.61 to 0.90), 1-day neonatal mortality by 42% (RR 0.58, 95% CI 0.42 to 0.82), 7-day neonatal mortality by 18% (RR 0.82, 95% CI 0.73 to 0.93), 28-day neonatal mortality by 14% (RR 0.86, 95% CI 0.65 to 1.13) and perinatal mortality by 18% (RR 0.82, 95% CI 0.74 to 0.91).

**Conclusions:**

Findings of this review show that implementation of NRT improves neonatal and perinatal mortality. Further good quality randomised controlled trials addressing the role of NRT for improving neonatal and perinatal outcomes may be warranted.

**Trial registration number:**

PROSPERO 2016:CRD42016043668

**What is already known?**

- A quarter of global neonatal deaths are due to birth asphyxia. The majority of these deaths occur in low-resource settings and are preventable.
- Neonatal resuscitation training (NRT) of birth attendants using mannequins result in improved knowledge and skills needed for resuscitation.
- Translation of NRT into improved neonatal outcomes and the effect estimates of improvements need to be re-evaluated and updated.

**What this study adds?**

- This meta-analysis assessed the impact of NRT on stillbirths, 1-day neonatal mortality, 7-day neonatal mortality, 28-day neonatal mortality and perinatal mortality.
- NRT resulted in significant reduction in stillbirths and early neonatal mortality. However, continuum of care is needed for mortality reduction from day 7 to 28.
- Future studies also need to establish the best combination of settings, trainee characteristics and training frequency to sustain the existing effect on perinatal mortality reduction.

## Introduction

Approximately a quarter of f million neonatal deaths worldwide are as a result of birth asphyxia.^1^ A large majority of these deaths occur in low-resource settings and are preventable. Approximately 5%–10% of newborns require some support to adapt to the extrauterine environment and to establish regular respiration.^1 2^ Simple resuscitative measures are often enough to resuscitate newborns that may even appear to be lifeless at birth. Studies have shown that essential newborn care has been effective in reducing stillbirths (SB).^3^

In developing countries, measures to improve resuscitative efforts through training of basic steps of neonatal resuscitation are expected to reduce birth asphyxia and neonatal mortality. Numerous studies have suggested that imparting neonatal resuscitation training (NRT) to healthcare providers involved in delivery process and handling of newborns has the potential to save newborn lives in low-income and middle-income settings^4–10^

Improvements in knowledge and skills of trainees following training programme in resource-limited settings have been reviewed. However, the impact on perinatal mortality outcomes has not been updated in last 5 years.^9^ The effect estimates of mortality reduction as a result of training of healthcare providers involved in delivery process and handling of newborns needs to be updated to inform hospital administrators and policy-makers the importance of investing in NRT to sustain and improve neonatal survival. A previous systematic review and meta-analysis^11^ assessed knowledge, skills, neonatal morbidity, neonatal mortality in first 7 days after birth and from day 8 to 28. However, it did not include outcomes of stlillbirth, 1-day neonatal mortality or perinatal mortality which has been included in our review.

The objective of this review is to assess the impact of NRT programme in reducing stillbirths, 1-day neonatal mortality, 7-day neonatal mortality, 28-day neonatal mortality and perinatal mortality.

## Materials and methods

### Inclusion criteria

#### Types of studies

We included relevant randomised, quasi-randomised controlled trials, interrupted time series studies and before–after studies regardless of language or publication status.

#### Types of participants (population) trained

We considered studies where NRT was provided to healthcare providers (including neonatologists, physicians, nurses, interns, midwives, traditional/community birth attendants, auxillary nurse midwives, village health workers, paramedics) involved in delivery process and handling of newborns in a community (home-based, rural and village clusters) or a hospital (including district hospitals, health centres, dispensaries, teaching/university hospitals, regional hospital, delivery/health centres, local hospitals and tertiary care hospital) setting.

#### Types of interventions and comparison

Studies in which any NRT was compared with a control group (that received no NRT) or compared with data before the study (pre-NRT vs post-NRT) were included. For this purpose, we considered any NRT programme of healthcare professionals, including the American Academy of Pediatrics’ (AAP) Neonatal Resuscitation Program (NRP), Helping Babies Breathe (HBB) or any other training programme that had NRP or HBB as a clearly mentioned component of training methodology.

#### Types of outcomes measures

We included following outcomes in the review:

1. Stillbirths: defined as number of deaths prior to complete expulsion or extraction of products of conception from its mother.
2. Fresh stillbirth: clinically defined as those deaths with no signs of life at any time after birth and without any signs of maceration.
3. 1-day neonatal mortality: defined as number of deaths in first 24 hours of life
4. 7-day neonatal mortality: defined as number of deaths in first 7 days of life
5. Perinatal mortality: defined as number of stillbirths and deaths in the first week of life.
6. 28-day neonatal mortality: defined as number of deaths in the first 28 days of life.

### Search strategy

We searched following electronic databases from inception to July 2016: MEDLINE (PubMed), The Cochrane Central Register of Controlled Trials (CENTRAL, The Cochrane Library); Education Resources Information Centre (ERIC), Web of Science, Science Citation Index and Scientific Electronic Library Online. The search strategies for PubMed and CENTRAL can be found in supplementary files S1 and S2 respectively. We also searched for ongoing trials at www.clinicaltrials.gov and www.controlled-trials.com. We searched published abstracts of conferences and examined bibliographies of retrieved articles for additional studies. We contacted and requested experts and authors in this field to provide possible unpublished work.

### Study selection and data extraction

#### Screening of studies

Two reviewers (MNK and AB) independently examined studies identified by literature search; discarded articles that did not fulfil the inclusion criteria and assessed full texts of all relevant articles for inclusion. A third reviewer (AP) resolved disagreement among the primary reviewers.

#### Data extraction and management

For all studies that fulfilled the inclusion criteria, two reviewers (KK, SB) extracted data (table 1 and 2). Third review author (AP) cross-checked the data and resolved discrepancies. For studies where required data was lacking or could not be calculated, we requested the corresponding author for details.

**Table 1:** Characteristic of included studies

Sr. No.	Author	Country	Study design	Study period	Funding
1	Bang *et al*^20^	India	RCT	36 months (1995–1998)	Ford Foundation USA The John D & Catherin T MacArthur Foundation USA
2	Ariawan et al* 8	Indonesia	Pre–Post training	NR	NR
3	Carlo *et al*^17^**	Argentina, Democratic Republic of Congo, Guatemala, India, Pakistan and Zambia	Pre–Post training and RCT	42 months (ENC: Mar 2005 and Feb 2007; NRP: Jul 2006–Aug 2008)	NICHD, Global Network for Women’s and Children’s Health Research Bill & Melinda Gates Foundation
4	Carlo *et al* 18	Argentina, Democratic Republic of Congo, Guatemala, India, Pakistan and Zambia	Pre–Post training and RCT	42 months (ENC: Mar 2005 and Feb 2007; NRP: Jul 2006–Aug 2008)	NICHD, Global Network for Women’s and Children’s Health Research, Bill & Melinda Gates Foundation
5	Gill *et al*^21^	Zambia	Prospective, cluster randomised and controlled effectiveness study	30 months (Jun 2006–Nov 2008)	Boston University and The Office of Health and Nutrition of The United State Agency for International Development AAP Unicef
6	Zhu *et al*^26^	China	Perspective study, pre–post training (traditional resuscitation vs NRPG)	24 months (1993–1995)	NR
7	Deorari *et al*^24^	India	Pre–post training (	NR	Laerdal Foundation Norway
8	Jeffery *et al*^28^	Macedonia	Pre–Post training	60 months (1997–2001)	International Project Unit, Ministry of Health, Macedonia IDA Credit, World Bank
9	Vakrilova *et al*^30^	Bulgeria	Pre–Post training (	48 months (2000–2003)	NR
10	O’Hare *et al*^25^	Uganda	Pre–Post training (historic group vs NRP pilot)	1 month (Dec 2001–Jan 2002)	Child Advocacy International
11	Opiyo *et al*^19^	Kenya	Pre–Post training	NR	Laerdal Foundation for Acute Medicine Wellcome Trust Senior Research Fellowship Award
12	Boo^31^	Malaysia	Pre–Post training, prospective observational study	100 months (Sep 1996–Dec 2004)	Perinatal Society of Malaysia
13	Sorensen *et al*^29^	Tanzania	Prospective study, Pre–Post training	14 weeks (Jul 2008–Nov 2008)	Danish Society of Obstetrics and Gynecology
14	Hole *et al*^32^	Malawi, Africa	Pre–Post training	30 months (Jun 2007–Dec 2009)	Stanford University School of Medicines, Medical Scholars Research Program Department of Community Relations at Lucil Packard Children’s Hospital
15	Msemo *et al*^22^	Tanzania	Pre–Post training	30 months (2009–2013)	AAP Laerdal Foundation for Acute Medicine
16	Goudar *et al*^23^	India	Pre–Post training (pretraining vs post HBB)	12 months (Oct 2009–Sep 2010)	AAP Global Implementation Task Force HBB Program, Laerdal Foundation for Acute Medicine, Stavanger Norway
17	Vossius *et al*^77^	Tanzania	Pre–Post training (pretraining vs post HBB)	24 months (Feb 2010–Jan 2012)	Laerdal Foundation for Acute Medicine and Municipality of Stavanger Norway Research Department of HLH, Tanzania
18	Ashish et al***	Nepal	Pre–Post training (pretraining vs post HBB)	15 months (Jul 2012–Sep 2013)	Laerdal Foundation for Acute Medicine Swedish Society of Medicine
19	Bellad *et al*^27^	Kenya, India (Belgaum, Nagpur)	Pre–Post training (pretraining vs post HBB)	24 months (Nov 2011–Oct 2013)	NORAD Laerdal Foundation and NICHD
20	Patel et al***	India (Nagpur)	Pre–Post training (pre-training vs post HBB)	24 months (Nov 2011–Oct 2013)	NORAD Laerdal Foundation and NICHD

*Data for this study has been taken from Lee *et al*^8^.

**Data for very low birth weight (<1500 g).

***Unpublished data obtained via personal communication with the author

AAP, American Academy of Pediatrics; ENC, essential newborn care; HBB, helping babies breathe; NICHD, National Institute of Child and Human Development; NR, not reported; NRPG, Neonatal Resuscitation Program Guidelines; RCT, randomised control trial.

#### Assessment of risk of bias in included studies

Two authors (SB, KK) independently assessed risk of bias for each study using criteria suggested by Cochrane Effective Practice and Organization of Care (EPOC)^12^ and using criteria outlined in Chapter 8 of Cochrane Handbook for Systematic Reviews of Interventions.^13^ Disagreements were resolved by discussion with the third reviewer (MNK).

### Data analysis

#### Measures of treatment effect

We conducted meta-analysis and reported pooled statistics as risk ratios (RR) with 95% confidence interval (CIs) for dichotomous data. We followed recommendations of the Cochrane Handbook for Systematic Reviews of Interventions Sections 9.2 and 9.4 for measuring the effects.^13^

#### Assessment of heterogeneity

We assessed heterogeneity amongst studies by inspecting forest plots for the overlap of confidence intervals, analysed statistical heterogeneity through Χ^2^ test (P value >0.10) and quantified through I^2^ statistics(Chapter 9.5 of Cochrane Handbook for Systematic Reviews).^13^ We regarded heterogeneity as substantial if in the Χ^2^ test for heterogeneity there was either I^2^>50%, or P value <0.10. We interpreted I^2^ values between 0% and 40% as possibly unimportant, 30% and 60% as possibly significant, 50% and 90% as possibly substantial and 75% and 100% as possibly considerable.

#### Assessment of reporting bias

We used funnel plots for assessment of publication bias if ten or more studies were included in a meta-analysis.

#### Data synthesis and analysis

We analysed the data using Review Manager V.5.3 software.^14^ We conducted meta-analyses for individual studies and reported pooled statistics as relative risk (RR) between experimental and control groups with 95% CI. We explored possible clinical and methodological reasons for heterogeneity, and in the presence of significant heterogeneity, we carried out sensitivity analysis and employed inverse-variance method with Random-effects model. We did not pool randomised and non-randomised (pre–post NRT) studies in the same meta-analysis.

#### Summary of findings table

We created ‘summary of findings’ (SoF) table using five GRADE considerations (study limitations, consistency of effect, imprecision, indirectness and publication bias) to assess the quality of a body of evidence. We used methods and recommendations described in Chapter 12 of the Cochrane Handbook for Systematic Reviews of Interventions^13^ using GRADEpro software.^15^ GRADE working Group grades of evidence were used in the SoF.^16^

## Results

### Search results

**Figure 1:**
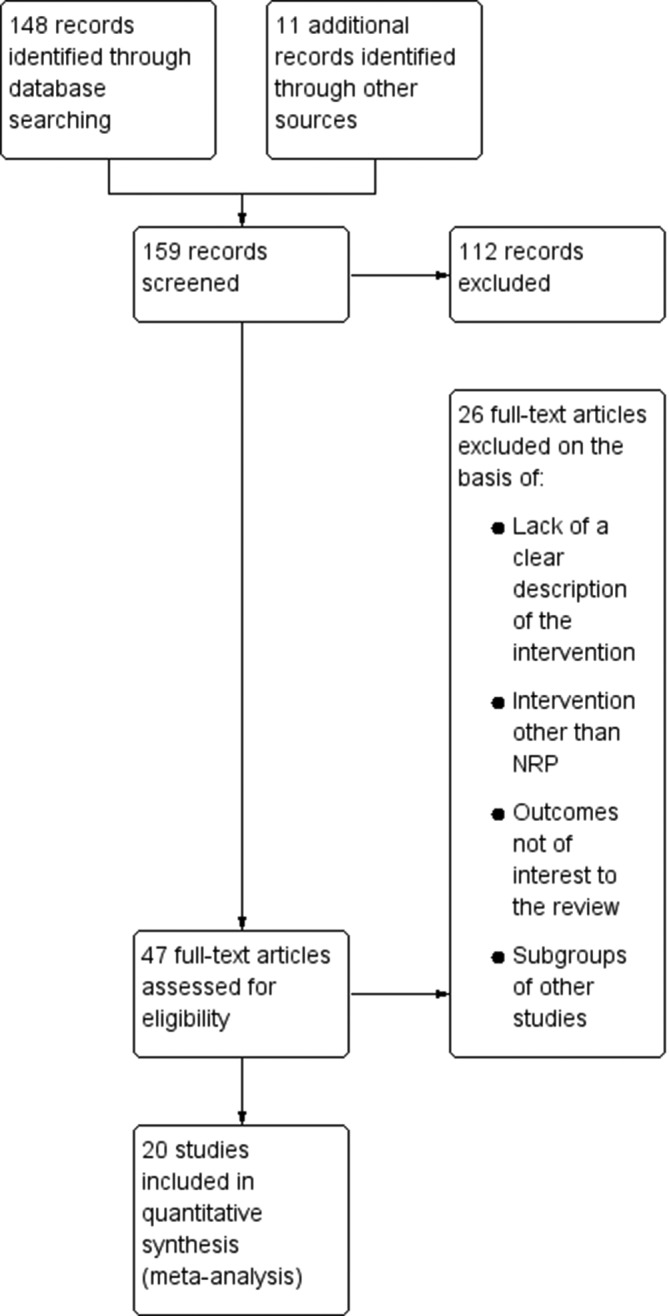
Flow diagram of the study selection process. NRP, Neonatal Resuscitation Program.

We identified 148 records through database searching and 11 records through other sources. After initial screening on the basis of title and abstract, we assessed 47 full-text articles for eligibility and finally included 20 articles in the meta-analysis. The screening details are presented in a Preferred Reporting Items for Systematic Reviews and Meta-Analyses flow diagram (figure 1).

### Included studies

Amongst included studies, two randomised trials addressed the efficacy of NRT in improving neonatal and perinatal outcomes, whereas 18 were pre–post studies. A full description of each study is included in table 1 and 2. All studies were from low-income and middle-income countries. Four studies were done in community setting, whereas 16 studies were carried in hospital setting.

**Table 2:** Characteristic of included studies (training and outcomes)

Sr. No.	Author	Training						**No. of births** A: control/pre B: intervention/post	Outcomes	**Criteria for delivery outcomes** A: inclusion B: exclusion
		Duration	Training setting	Type	Trainers	Trainees	Assessment			
1	Bang *et al*^20^	NR	Community (86 villages)	A package of home-based neonatal care, health education including ENC Suction, stimulation Artificial respiration by mouth to mask and tube and mask	NR	Community birth attendants Village health workers	NR	A: 1159 B: 1005	1. SB 2. NMR: day 7 3. Perinatal mortality	A: NR B: NR
2	Ariawan et al* 8	NR	Community	NRT including Use of tube mask Refresher training at 3, 6 and 9 months, use of video Post resuscitation care	NR	Midwives	NR	A: 9816 B: 16 053	1. SB 2. NMR: day 28	A: NR B: NR
3	Carlo *et al*^17^**	3 days	Rural communities (7 sites in 6 countries for ENC; 88 for NRP)	ENC sensitisation followed by in-depth NRT including Initial resuscitation steps BMV	AAP-trained trainer Research staff, either a physician or nurse	Community birth attendants	NR	A: 359 B: 273	1. SB 2. FSB 3. NMR: day 7 4. PNMR	A: BW <1500 g B: NR
4	Carlo *et al*^18^	3 days	Rural communities (7 sites in six countries for ENC; 88 for NRP)	ENC sensitisation followed by in-depth NRT including Initial resuscitation steps BMV	AAP-trained trainer Research staff, either a physician or nurse	Community birth attendants	NR	A: 35 017 B: 29 715	1. SB 2. FSB 3. NMR: day 1 4. NMR: day 7 5. PNMR	A: BW >1500 g B: NR
5	Gill *et al*^21^	2 weeks	Community (rural district setting)	NRT modified from AAP/AHA including Initial steps PPV Use of manikins to demonstrate and practice skills	NR	60 Community birth attendants/TBAs	One to one skills assessment	A: 1536 B: 1961	1. SB 2. NMR: day 7 3. NMR: day 28 4. PNMR	A: NR B: NR
6	Zhu *et al*^26^	NR	Hospital (1 hospital)	NRPG curriculum established from AAP and AHA including Suction BMV or ET ventilation Intubation	NR	Hospital birth attendants	NR	A: 1722 B: 4751	1. NMR: day 1 2. NMR: day 7	A: NR B: NR
7	Deorari *et al*^24^	NR	Hospital (14 teaching hospitals)	AAP/AHA-modified NRT with ToT approach	2 Faculty member trainer per facility	Hospital-based birth attendants	No skills assessment	A: 7070 B: 25 713	1. NMR: day 28	A: NR B: NR
8	Jeffery *et al*^28^	9 weeks	Hospital (3 tertiary care, 13 district hospitals)	A package of perinatal practices with NRT	Australian-trained Macedonian teachers (doctors and nurses)	Doctors and nurses	MCQ, SAQ and OSCE (practical test)	A: 69 840 B: 45 458	1. SB 2. NMR: day 7 3. PNMR	A: NR B: NR
9	Vakrilova *et al*^30^	NR	Hospital (delivery rooms of city hospitals)	French–Bulgarian Program on NRT	NR	Neonatologist Obstetrician Midwives	NR	A: 67 948 B: 67 647	1. NMR: day 7	A: NR B: NR
10	O’Hare *et al*^25^	10 days training (5 days classroom+5 days delivery suite)	Hospital (1 teaching hospital)	NRT including Airway management BMV Cardiac massage Use of manikins to demonstrate and practice skills	NR	5 members of nursing staff	NR	A: 1296 B: 1046	1. SB	A: NR B: NR
11	Opiyo *et al*^19^	1 day	Hospital (1 maternity hospital)	NRT including Initial steps BMV (use of bag valve mask device) CC Use of manikins to demonstrate and practice skills	Instructor completed Kenya Resuscitation Council Advanced Life Support Generic Instructor Course	Nurse/midwives	MCQ and formal test scenario evaluating skills	A: 4084 B: 4302	1. SB 2. NMR: day 28	A: NR B: NR
12	Boo^31^	NR	Hospital	AAP-NRT tailored to local needs including Initial steps BMV CC ET ToT approach, a national-level training programme	37 Core instructors Doctors and nurses	14 575 Doctors Nurses Medical assistants Medical students	Written and practical test	A: 541 721 B: 465 140	1. SB 2. NMR: day 28 3. PNMR	A: NR B: NR
13	Sorensen *et al*^29^	2 days	Hospital (1 referral hospital)	ALSO a widespread EmONC Use of manikins to demonstrate and practice skills	NR	High-level and mid-level staff involved in delivery	NR	A: 577 B: 565	1. SB	A: BW >1000 g B: Missing data
14	Hole *et al*^32^	1 day	Hospital (1 university hospital and 1 referral hospital)	AAP modified NRT to include Initial steps BMV CC and special consideration Use of manikins to demonstrate and practice skills	Paediatrics residents from Stanford University	Physician Clinical officers Midwives	Survey covering knowledge, skills and attitude	A: 3449 B: 3515	1. NMR: day 28	A: NR B: NR
15	Msemo *et al*^22^	1 day	Hospital (3 referral hospitals, 4 regional hospitals and 1 district hospital)	HBB training including Stimulation Suctioning Face and mask ventilation ToT approach Use of simulators for hands on practice FBOS training—reported by 1 site	40 Trainers	Hospital birth attendants	Practical test	A: 8124 B: 78 500	1. SB 2. FSB 3. NMR: day 1	A: BW >750 g for live birth BW >1000 g for FSB
16	Goudar *et al*^23^	1 day	Hospital (primary health centres and rural and urban hospitals)	HBB–AAP-based NRT Initial steps Stimulation Suctioning BMV ToT model Paired teaching Use of manikins to demonstrate and practice skills	18 Master trainers trained by AAP Physicians and nurses	599 Birth attendants	Written and verbal MCQ, BMV by demonstration—OSCE	A: 4187 B: 5411	1. SB 2. FSB 3. NMR: day 28	A: GA >28 wks B: NR
17	Vossius *et al*^77^	1 day	Hospital (1 tertiary hospital)	HBB–AAP-based NRT including BNC and resuscitation Simulation-based training using manikins ToT approach	40 Master trainers	Hospital-based birth attendants	Knowledge and technical skills	A: 4876 B: 4734	1. FSB 2. NMR: day 7	A: NR B: NR
18	Ashish et al***	2 days	Hospital (1 tertiary hospital)	HBB–AAP-based NRT with QIC; train the trainer model, paired teaching Skills and practice ToT model Use of manikins to demonstrate and practice skills	NR	Obstetricians Anaesthesiologist Medical doctors Students Nurse/midwives	NR	A: 9588 B: 15 520	1. SB 2. FSB 3. NMR: day 1 4. PNMR	A: GA >22 wks B: NR
19	Bellad *et al*^27^	3 days	Hospital (39 primary, 21 secondary and 11 tertiary facilities)	HBB–AAP-based NRT including Initial steps Stimulation, suctioning BMV Refresher training QI activities ToT model Paired teaching Use of manikins to demonstrate and practice skills	Neonatologists Paediatricians Obstetricians Nurses	Hospital-based birth attendants Paediatricians Obstetricians Physicians Residents Nursing staff Medical assistants	MCQ, OSCE for skills assessment	A: 15 232 B: 15 985	1. FSB 2. NMR: day 1 3. NMR: day 7 4. NMR: day 28 5. PNMR	A: BW >1500 g B: BW unknown,<1500, >5500 and MSB
20	Patel et al***	3 days	Hospital (2 primary, 4 secondary HTML validation and 7 tertiary facilities)	HBB–AAP-based NRT including Initial steps Stimulation, suctioning BMV Refresher training and QI activities ToT model Paired teaching Use of manikins to demonstrate and practice skills	Neonatologists Paediatricians Obstetricians Nurses	eHospital-based birth attendants Paediatricians Obstetricians Physicians Residents Nursing staff Medical assistants	MCQ, OSCE for skills assessment	A: 38 078 B: 40 870	1. SB 2. FSB 3. NMR: day 1 4. NMR: day 7 6. PNMR	A: GA >20 wks B: NR

*Data for this study has been taken from Lee *et al*^8^.

**Data for very low-birth weight (<1500 g).

***Unpublished data obtained via personal communication with the author

AAP, American Academy of Pediatrics; AHA, American Heart Association; ALSO, Advanced Life Support in Obstetrics; BMV, bag and mask ventilation; BW, birth weight; CC, chest compression; EmONC, Emergency Obstetrics & Neonatal Care; ENC, essential newborn care; ET, endotracheal tube; FBOS, frequent brief onsite simulation; FSB, fresh stillbirth; GA, gestational age, HBB, helping babies breathe; MCQ, multiple choice questions; NICHD, National Institute of Child and Human Development; NMR, neonatal mortality rate; NORAD, Norwegian Agency for Development Cooperation; NR, not reported; NRPG, Neonatal Resuscitation Program Guidelines; NRT, neonatal resuscitation training; OSCE, objective structured clinical evaluation; PNMR, perinatal mortality rate; PPV, positive pressure ventilation; QI, quality improvement; QIC, quality improvement cycle; RCT, randomised control trial; SAQ, short answer questions; SB, all stillbirth; TBA, traditional birth attendants; ToT, training of trainer; wks, weeks.

Carlo *et al*^17 18^ assessed baseline perinatal outcomes, then imparted Essential Newborn Care (ENC) training to all which also included basic steps of NRT. They then randomised all clusters that had received ENC training into two groups. One group received an in-depth NRT while the other group did not (control group). For this study we evaluated the pre-ENC outcome of all clusters and compared them to outcomes of those clusters that received ENC +post ENC in-depth NRT. We therefore did not include this study in the NRT versus control analysis because the control group had also received NRT as a part of ENC training.

The study from Kenya had a complex design of randomisation of health workers to two groups—early training (phase I) or late training (phase II) and did not include a control group without training.^19^ Therefore, we analysed this study as before–after study where the rate of stillbirths prior to any training were compared with the rate of stillbirths after all phases of training.

Participants of the NRT programme differed across studies and included village health workers, community birth attendants,^17 18 20^ community birth attendants/traditional birth attendants,^21^ hospital-based birth attendants,^19 22–26^ or hospital-based birth attendants including high-level and mid-level staff/specialists.^27–34^

Different types of training employed by studies included AAP, HBB or NRP curricula^23 24 27 31 32 34 35^ AAP/American Heart Association (AHA),^21 24 26^ basic neonatal resuscitation and ENC,^17–19 25^ home-based neonatal care, basic training with mouth to mask or tube and mask resuscitation,^35^ Advanced Life Support in Obstetrics (ALSO),^29^ Bulgarian program on NRT.^30^ The duration of NRT also differed acrossstudies.

We also included two unpublished trials after permission from authors (tables 1 and 2).

### Excluded studies

Studies that included interventions that did not qualify as NRT were excluded from the review. These included trainings in safe birthing techniques,^36^ Emergency Obstetric and Neonatal Care (EmONC),^37 38^ ENC,^39–41^promotion of antenatal care and maternal health education,^42^and newborn care intervention package.^43^

Other interventions that did not qualify as NRT^44–50^ or included interventions like neonatal intensive care unit/special neonatal care unit training^51 52^ were also excluded.

Studies in which desired outcomes (fetal and neonatal outcome) were not assessed,^53–58^ or only trainees/training outcomes were assessed,^59–73^ were also excluded from the analysis.

Some studies that were subgroups of larger studies like Ersdal *et al*.^74 75^ (subgroup of Msemo *et al*^22^), Matendo *et al*^76^(subgroup of Carlo *et al*^18^), Matendo *et al*^76^ and Vossius *et al*^77^ (subgroup of Msemo *et al*^22^) were also not included. However, Vossius *et al*^77^ was included in the analysis for outcomes where data from^22^ Msemo *et al*^22^ were not available.

Risk of bias in included studies has been depicted in table 3.

**Table 3:** Risk of bias assessment across studies

	Bang *et al*^20^	Carlo *et al*^17^	Carlo *et al*^18^	Gill *et al*^21^	Zhu *et al*^26^	Deorari *et al*^24^	Jeffery *et al*^28^	O’Hare *et al*^25^	Opiyo *et al*^19^	Boo^31^	Sorensen *et al*^29^	Hole *et al*^32^	Msemo *et al*^22^	Goudar *et al*^23^	Vossius *et al*^77^	Ashish *et al (Unpublished data)*	Bellard *et al*	Patel *et al (Unpublished data)*
Adequate sequence generation?	High risk			Low risk														
Allocation concealment?	High risk			Low risk														
Incomplete outcome data addressed?	High risk	Low risk	Low risk	Low risk	Unclear risk	Unclear risk	Unclear risk	Low risk	Unclear risk	Low risk	Low risk	High risk	Unclear risk	Unclear risk	Low risk	Low risk	Low risk	Low risk
Free of selective reporting?	Low risk	Low risk	Low risk	Low risk	Low risk	Low risk	Low risk	Low risk	Low risk	Low risk	Low risk	Low risk	Low risk	Low risk	Low risk	Low risk	Low risk	Low risk
Free of other bias?	Unclear risk	Low risk	Low risk	Low risk	Low risk	Low risk	Low risk	Unclear risk	Unclear risk	Uncleat risk	Low risk	Unclear risk	Low risk	Unclear risk	High risk	Low risk	High risk	Unclear risk
Baseline outcomes similar?		Low risk	Low risk		Unclear risk	Unclear risk	Unclear risk	Unclear risk	unclear risk	Uncleat risk	Unclear risk	Unclear risk	Unclear risk	Unclear risk	Unclear risk	Unclear risk	Unclear risk	Unclear risk
Free of contamination?		Low risk	Low risk		Low risk	Low risk	Unclear risk	Low risk	Low risk	High risk	Low risk	High risk	Low risk	Low risk	High risk	Low risk	Low risk	Low risk
Baseline characteristics similar?		Unclear risk	Unclear risk		Unclear risk	Unclear risk	Unclear risk	Unclear risk	Unclear risk	Low risk	Low risk	Unclear risk	Unclear risk	Low risk	Unclear risk	High risk	Low risk	Low risk

### Effects of interventions

Neonatal and perinatal outcomes were reported in majority of included studies. The overall analysis showed a trend towards reduction in neonatal deaths, early neonatal deaths, perinatal deaths and stillbirths with NRT; most of which are statistically significant.

### NRT verses control

**Figure 2:**
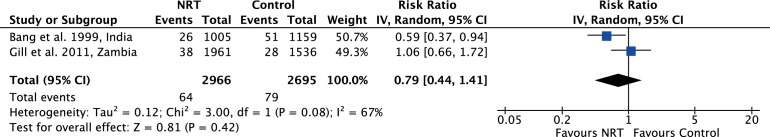
Forest plot comparing all SB between the NRT and the control groups. NRT, neonatal resuscitation training; SB, stillbirths.

**Figure 3:**
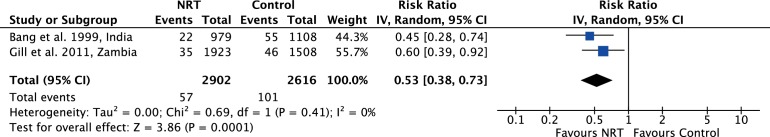
Forest plot comparing 7-day neonatal mortality between the NRT and the control groups. NRT, neonatal resuscitation training.

**Figure 4:**
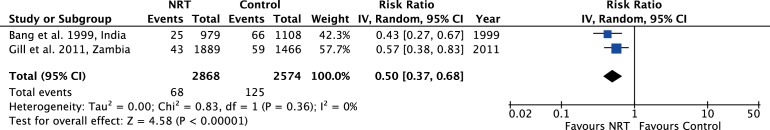
Forest plot comparing 28-day neonatal mortality between the NRT and the control groups. NRT, neonatal resuscitation training.

**Figure 5:**
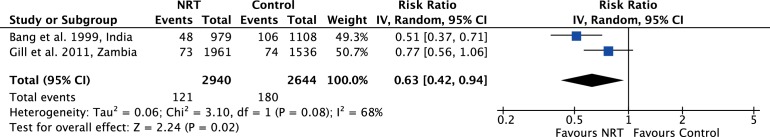
Forest plot comparing perinatal mortality between the NRT and the control groups. NRT, neonatal resuscitation training.

The meta-analysis for NRT verses control shows that NRT decreases the risk of all stillbirths by 21% (RR 0.79, 95% CI 0.44 to 1.41; participants=5661; studies=2; I^2^=67%) (figure 2), 7-day neonatal deaths by 47% (RR 0.53, 95% CI 0.38 to 0.73; participants=5518; studies=2; I^2^=0%) (figure 3), 28-day neonatal deaths by 50% (RR 0.50, 95% CI 0.37 to 0.68; participants=5442; studies=2; I^2^=0%) (figure 4), and perinatal deaths by 37% (RR 0.63, 95% CI 0.42 to 0.94; participants=5584; studies=2; I^2^=68%)(figure 5). The effect was significant for ay 7-day neonatal mortality, 28-day neonatal mortality and perinatal mortality. Significant heterogeneity was observed in analysis of total stillbirths and perinatal mortality.

The grade of quality of evidence for the meta-analysis of the trials was moderate to high (table 4).

**Table 4:** Summary of findings for NRT versus control groups

Outcomes	Anticipated absolute effects (95% CI) – risk with no NRP	Anticipated absolute effects (95% CI) – risk with NRP	Relative effect (95% CI)	No of participants (studies)	Quality of the evidence (GRADE)
All stillbirth	29 per 1000	23 per 1000 (13 to 41)	RR 0.79 (0.44 to 1.41)	5661 (2 RCTs)	⨁◯◯◯ Very low*†
Fresh stillbirth	Outcome not reported	Outcome not reported	Outcome not reported	Outcome not reported	⨁◯◯◯ Very low‡
1-day neonatal mortality	Outcome not reported	Outcome not reported	Outcome not reported	Outcome not reported	⨁◯◯◯ Very low‡
7-day neonatal mortality	39 per 1000	20 per 1000 (15 to 28)	RR 0.53 (0.38 to 0.73)	5518 (2 RCTs)	⨁⨁⨁⨁ High
28-day neonatal mortality	49 per 1000	24 per 1000 (18 to 33)	RR 0.50 (0.37 to 0.68)	5442 (2 RCTs)	⨁⨁⨁⨁ High
Perinatal mortality	68 per 1000	43 per 1000 (29 to 64)	RR 0.63 (0.42 to 0.94)	5584 (2 RCTs)	⨁⨁⨁◯ Moderate§

*I^2^ is 67% and the two trials were inconsistent in the direction of effect. Quality of evidence downgraded by two for inconsistency and imprecision (figure 2).

†The 95% CI of the pooled estimate includes null effect. Quality of evidence downgraded by one for imprecision (figure 2).

‡No evidence to support or refute.

§Though I^2^ is 68%, the 95% CI of the pooled estimate does not include the null effect. Quality of evidence downgraded by one for inconsistency (figure 5).

NRT, neonatal resuscitation training; RCTs, randomised controlled trial; RR, risk ratio.

### Post-NRT verses pre-NRT

**Figure 6:**
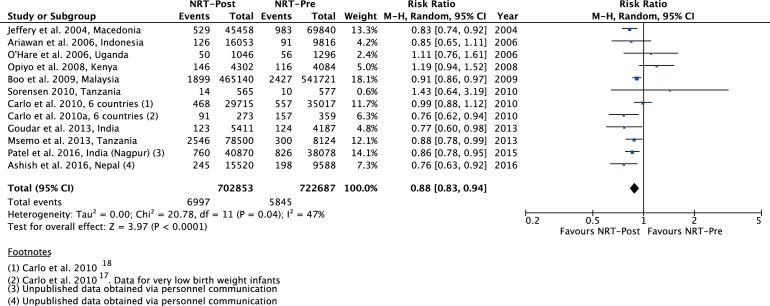
Forest plot comparing all SB between the post-NRT and the pre-NRT groups. NRT, neonatal resuscitation training; SB, stillbirths.

**Figure 7:**
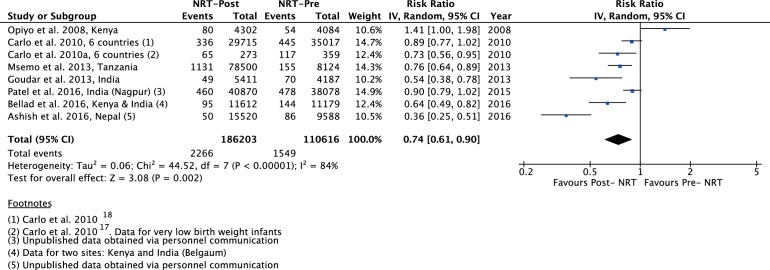
Forest plot comparing fresh SB between the post-NRT and the pre-NRT groups. NRT, neonatal resuscitation training; SB, stillbirths.

**Figure 8:**
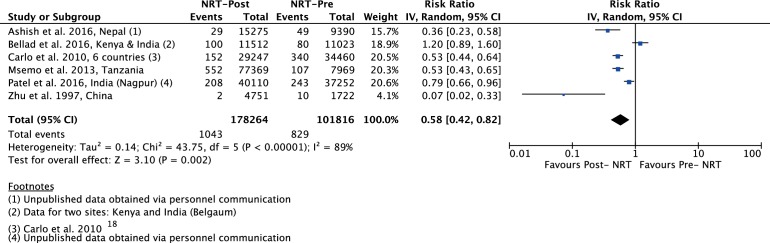
Forest plot comparing 1-day neonatal mortality between the post-NRT and the pre-NRT groups. NRT, neonatal resuscitation training.

**Figure 9:**
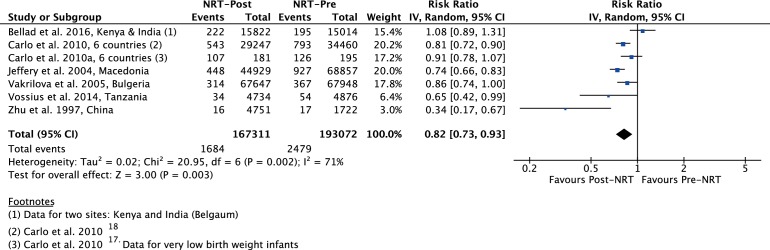
Forest plot comparing 7-day neonatal mortality between the post-NRT and the pre-NRT groups. NRT, neonatal resuscitation training.

**Figure 10:**
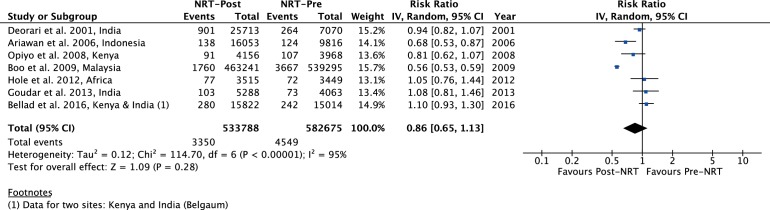
Forest plot comparing 28-day neonatal mortality between the post-NRT and the pre-NRT groups. NRT, neonatal resuscitation training.

**Figure 11:**
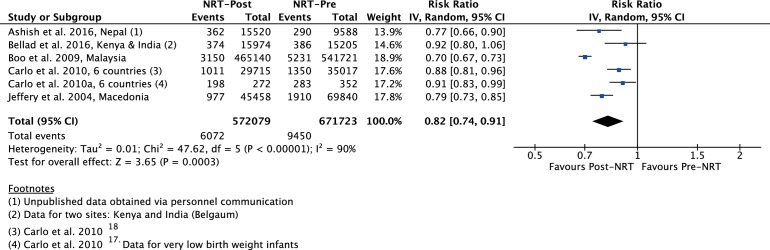
Forest plot comparing perinatal m between the post-NRT and the pre-NRT groups. NRT, neonatal resuscitation training.

**Figure 12:**
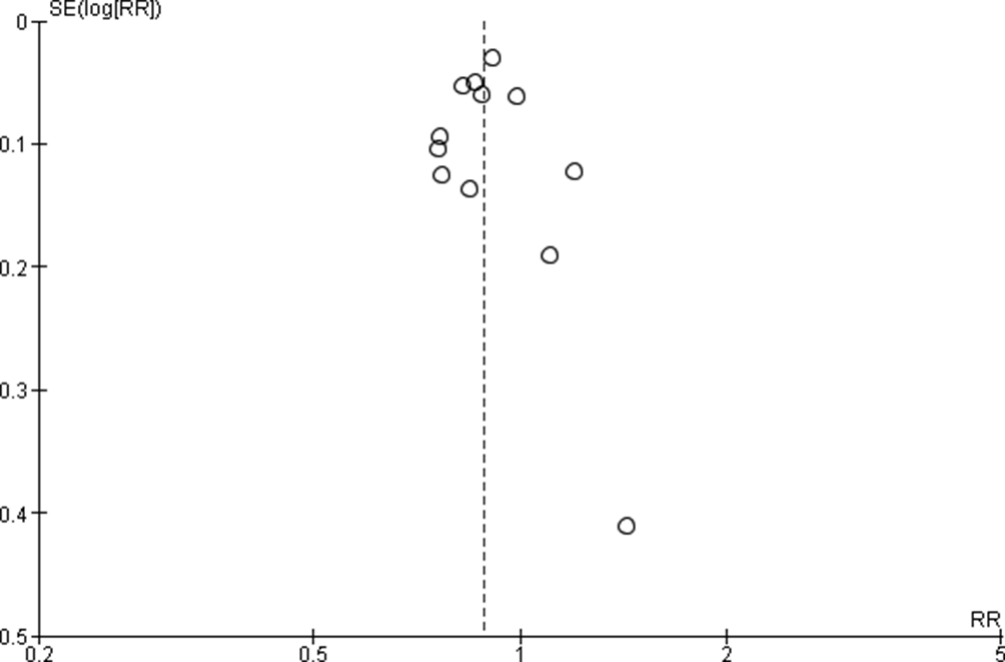
Funnel plot of comparison: Post-NRT verses pPre-NRT for all SB. NRT, neonatal resuscitation training; RR, risk ratio; SB, stillbirths.

The meta-analysis of post-NRT verses pre-NRT shows that post-NRT decreases the risk of all stillbirths by 12% (RR 0.88, 95% CI 0.83 to 0.94; participants=1 425 540; studies=12; I^2^=47%, figure 6), fresh stillbirths by 26% (RR 0.74, 95% CI 0.61 to 0.90; participants=296 819; studies=8; I^2^=84%, figure 7), 1-day neonatal mortality by 42% (RR 0.58, 95% CI 0.42 to 0.82; participants=280 080; studies=6; I^2^=89%, figure 8), 7-day neonatal mortality by 18% (RR 0.82, 95% CI 0.73 to 0.93; participants=360 383; studies=7; I^2^=71%, figure 9), 28-day neonatal mortality by 14% (RR 0.86, 95% CI 0.65 to 1.13; participants=1 116 463; studies=7; I^2^=95%, figure 10) and perinatal mortality by 18% (RR 0.82, 95% CI 0.74 to 0.91; participants=1 243 802; studies=6; I^2^=90%, figure 11). The changes were significant in all the outcomes; except 28-day neonatal mortality. Heterogeneity was significant in all outcomes except all stillbirths. We created a funnel plot for all stillbirths, which showed asymmetry, thereby indicating a publication bias (figure 12).

The quality of evidence for NRT verses control was very low for SB and 1-day neonatal mortality, high for 7-day and 28-day neonatal mortality and moderate for perinatal mortality (table 4). The quality of evidence for post-NRT verses pre-NRT was very low for all our outcomes (table 5).

**Table 5:** Summary of findings for Post-NRT versus Pre-NRT groups

Outcomes	Anticipated absolute effects (95% CI) Risk with pre-NRP	Anticipated absolute effects (95% CI) Risk with post-NRP	Relative effect (95% CI)	No of participants (studies)	Quality of the evidence (GRADE)
All stillbirths	8 per 1000	7 per 1000 (7 to 8)	RR 0.88 (0.83 to 0.94)	1 425 540 (12 observational studies)	⨁◯◯◯ Very low^*†‡^
Fresh stillbirths	15 per 1000	11 per 1000 (9 to 13)	RR 0.74 (0.61 to 0.90)	296 819 (8 observational studies)	⨁◯◯◯ Very low^*†§^
1-day neonatal mortality	8 per 1000	5 per 1000 (4 to 7)	RR 0.58 (0.42 to 0.82)	280 080 (6 observational studies)	⨁◯◯◯ Very low ^*¶^
7-day neonatal mortality	13 per 1000	11 per 1000 (9 to 12)	RR 0.82 (0.73 to 0.93)	360 383 (7 observational studies)	⨁◯◯◯ Very low ^*† **^
28-day neonatal mortality	8 per 1000	7 per 1000 (5 to 9)	RR 0.86 (0.65 to 1.13)	1 116 463 (7 observational studies)	⨁◯◯◯ Very low ^* ††^
Perinatal mortality	14 per 1000	12 per 1000 (10 to 13)	RR 0.82 (0.74 to 0.91)	1 243 802 (6 observational studies)	⨁◯◯◯ Very low ^* §§ ¶¶^

*Pre–post studies. Quality of evidence downgraded by one for risk of bias (table 1 and 2).

† Studies differ in the settings, type of NRP, duration and type trainees. Quality of evidence downgraded by one for indirectness (table 1 and 2).

‡ Publication bias detected in the funnel plot. Quality of evidence downgraded by one for publication bias (figure 12).

§ Although I^2^ is 84%, the effect estimates of all included studies do not differ in the direction of effect. Quality of effect downgraded by one for inconsistency (figure 7).

¶ Although I^2^ is 89%, the effect estimates of all the included studies (except Bellard et al.) do not differ in the direction of effect. Quality of effect downgraded by one for inconsistency (figure 8).

** Although I^2^ is 71%, the effect estimates of all the included studies (except Bellard et al.) do not differ in the direction of effect. Quality of effect downgraded by one for inconsistency (figure 9).

†† I^2^ is 95% and the effect estimates cross the life of no effect. Quality of evidence downgraded by two for inconsistency and imprecision (figure 10).

‡‡The effect estimate crosses the line of no effect. Quality of evidence downgraded by one for imprecision (figure 10).

§§ Although I^2^ is 90%, the effect estimates of all the included studies do not differ in the direction of effect. Quality of effect downgraded by one for inconsistency (figure 11).

¶¶ Studies differ in setting, type of NRP and trainees. Quality of evidence downgraded by one for indirectness (table 1 and 2).

NRP, Neonatal Resuscitation Program; NRT, neonatal resuscitation trainings; RR, risk ratio; SB, stillbirths.

## Discussion

This meta-analysis assessed the impact of any NRT programme either by itself or as a part of newborn care package on rates of stillbirths, perinatal mortality, all-cause neonatal mortality on day-1, up till day-7 and till 28th day after birth. We did not evaluate intrapartum-related neonatal deaths or asphyxia/cause-specific neonatal mortality. Mortality in neonates <7 days of life is a proxy measure for intrapartum-related deaths.^43 78^ Meta-analysis of before–after studies showed a significant reduction in all stillbirths by 12% (12 studies) and of FSB by 26% (8 studies). The reduction in fresh stillbirths can be attributed to NRT that helps in resuscitating neonates that appear lifeless at birth.^17 18^ Of 12 studies, seven studies reported a significant and one study reported a non-significant reduction in fresh stillbirths. However, a non-significant increase in risk of stillbirths was reported in three African studies which blunted the impact of NRT on reduction of stillbirths.

There was reduction in 1-day mortality of 42% (6 studies) and that of 7-day mortality was 18%. All studies included in the analysis (figures 8 and 9) showed a reduction with an exception of one study.^27^ Failure to observe reduction in mortality in Bellad *et al* could be due to two reasons. First, NRT was provided in diverse health systems within a short period of time. Second, mortality was not assessed in facilities where training was imparted but was measured in the population.

The meta-analysis showed a non-significant reduction of 14% in 28-day mortality. Of the seven included studies only two studies reported a significant reduction in mortality. Resuscitation at delivery helps to reduce neonatal mortality in the first hour of birth when the neonate is at the highest risk of intrapartum-related deaths^3^ and the impact diminishes subsequently. For reduction of 28-day neonatal mortality, post-resuscitation specialised care for survivors is required and only NRT is unlikely to have the desired impact on 28-day neonatal mortality.^79 80^

Trials that randomise facilities to NRT versus controls (where NRT is not a standard practice) would be ideal to assess the reduction in neonatal mortality. Trials are also likely to result in higher impact as compared with before–after studies as other changes at health facilities or in communities during the time period of before–after studies can confound the results. Because NRT is a standard practice and randomising individuals or clusters to no resuscitation training is unethical, there were only two trials available for the meta-analysis.^20 21^ They showed a reduction of 7-day neonatal mortality and 28-day mortality by 47% (figure 3) and 50% (figure 4), respectively. The perinatal mortality reduced by 37% (figure 5) with no significant reduction in SB rates.

Previously, an expert panel published a systematic review for community-based studies and conducted a meta-analysis that evaluated whether NRT reduced all-cause neonatal mortality in th first 7 days of life. They reported a 38% reduction in mortality which is larger than the 18% (7 studies) reduction observed in the current meta-analysis. Our meta-analysis included community-based studies that resulted in a smaller effect size. Community-based studies (trials or before–after) report a smaller reduction effect on any day neonatal mortality.^8 17 18 47^ The reduction in effect size of neonatal mortality in these studies can arise due to several reasons. All births in the intervention community may not be attended by birth attendants trained in neonatal resuscitation, especially if it is a home delivery.^81 82^ Second, women may decide to deliver at facilities or homes outside communities where NRT has been imparted. Finally, assessing mortality outcomes in the community can be challenging. Another meta-analysis^11^ was published in Cochrane which evaluated outcomes such as knowledge, skills, neonatal morbidity, neonatal mortality in first 7 days after birth and from day 8 to 28. This analysis did not include stillbirths, 1-day neonatal mortality or perinatal mortality that was included in the current meta-analysis.

The current meta-analysis consists largely of before–after studies with lack of concurrent control group that limits isolation of effect of resuscitation training alone from other changes at health facilities or in communities during the time period. Other limitation is lack of consistency of settings, duration of training, varying study designs and lack of consistent outcomes which contributed to substantial heterogeneity. Lack of subgroup analysis of type of health facilities may be perceived as a limitation. An improvement in mortality would be maximised in low-resource settings with poor quality of care. However, it is presumed that there is regular training of health workers in basic resuscitation skills in higher levels of care that would translate to higher quality of care. Our recent study^83 84^ that evaluated the knowledge and skills of trainees trained in HBB included 384 tertiary-level facilities in India. Only 3% of physicians and 5% of nurses were able to pass the pre-training bag and mask resuscitation skill assessment.^84^ Therefore, in the absence of reporting of pre-training skills of health workers in low-resource or high-resource settings or any indicator of quality of care, it would be erroneous to conduct a subgroup analysis based merely on resource settings and mostly will not change the results or the main message of this meta-analysis. We emphasise that despite the heterogeneity in settings, type of training, type of trainees, type of trainers and the duration of training, this study showed an improvement in mortality at and soon after birth.

To conclude, NRT resulted in reduction in stillbirths and improved survival of newborns. The impact on survival of newborns can be further improved by providing a continuum of care beyond 7 days which is not addressed by NRT alone.

The meta-analysis performed showed beneficial effect of NRT in improving neonatal and perinatal outcomes. The models of training were not consistent across studies, with variations in training, trainee and setting. Generalisation of results of the pooled analysis to many currently available programme may not be appropriate. There was evidence of heterogeneity across studies in our meta-analyses; however, overall there is consistency in the direction of effect.

This review identified several important limitations of the current evidence from included studies. Due to inadequate information about the methodology followed and variety of resuscitation programmes in included studies, the quality of the evidence was downgraded for risk of bias and indirectness resulting in inability to adequately assess the effects of this intervention.

## Conclusions

### Implications for practice

This review shows that the implementation of NRT improves neonatal and perinatal outcomes.

### Implications for research

Further good quality, multicentric randomised controlled trials addressing the role of NRT for improving neonatal and perinatal outcomes may be warranted. Impact of NRT on improving neonatal and perinatal outcomes as well as the best combination of settings and type of trainee should be established in future trials. More studies need to be done to assess the frequency with which NRT needs to be conducted to sustain the existing effect on perinatal mortality reduction.

## References

[1] David M, Mark D Obstetrics & Gynaecology: an evidence-based text for MRCOG. 3rd edition London, United Kingdom: Taylor Francis Ltd, 2010.

[2] Australian resuscitation council, New Zealand resuscitation council. The resuscitation of the newborn infant in special circumstances. ARC and NZRC guideline 2010. Emerg Med Australas 2011;23:445–7. doi:10.1111/j.1742-6723.2011.01442_15.x 21824309 10.1111/j.1742-6723.2011.01442_15.x

[3] Wall SN, Lee AC, Niermeyer S, et al Neonatal resuscitation in low-resource settings: what, who, and how to overcome challenges to scale up? Int J Gynaecol Obstet 2009;107(Suppl 1):S47–S64. doi:10.1016/j.ijgo.2009.07.013 19815203 10.1016/j.ijgo.2009.07.013 PMC2875104

[4] Palme-Kilander C Methods of resuscitation in low-apgar-score newborn infants-a national survey. Acta Paediatr 1992;81:739–44. doi:10.1111/j.1651-2227.1992.tb12094.x 1421875 10.1111/j.1651-2227.1992.tb12094.x

[5] Kattwinkel J, Niermeyer S, Nadkarni V, et al Resuscitation of the newly born infant: an advisory statement from the pediatric working group of the international liaison committee on resuscitation. Resuscitation 1999;40:71–88. doi:10.1016/S0300-9572(99)00012-X 10225280 10.1016/s0300-9572(99)00012-x

[6] International Liaison Committee on Resuscitation. The international liaison committee on resuscitation (ILCOR) consensus on science with treatment recommendations for pediatric and neonatal patients: neonatal resuscitation. Pediatrics 2006;117:e978–88. doi:10.1542/peds.2006-0350 16618791 10.1542/peds.2006-0350

[7] Sousa S, Mielke JG Does resuscitation training reduce neonatal deaths in low-resource communities? A systematic review of the literature. Asia Pac J Public Health 2015;27:690–704. doi:10.1177/1010539515603447 26378066 10.1177/1010539515603447

[8] Lee AC, Cousens S, Wall SN, et al Neonatal resuscitation and immediate newborn assessment and stimulation for the prevention of neonatal deaths: a systematic review, meta-analysis and Delphi estimation of mortality effect. BMC Public Health 2011;11:S12 doi:10.1186/1471-2458-11-S3-S12 10.1186/1471-2458-11-S3-S12 PMC3231885 21501429

[9] Reisman J, Arlington L, Jensen L, et al Newborn resuscitation training in resource-limited settings: a systematic literature review. Pediatrics 2016;138:e20154490 doi:10.1542/peds.2015-4490 27388500 10.1542/peds.2015-4490

[10] American heart association, american academy of pediatrics. 2005 American heart association (AHA) guidelines for cardiopulmonary resuscitation (CPR) and emergency cardiovascular care (ECC) of pediatric and neonatal patients: neonatal resuscitation guidelines. Pediatrics 2006;117:e1029–38. doi:10.1542/peds.2006-0349 16651282 10.1542/peds.2006-0349

[11] Dempsey E, Pammi M, Ryan AC, et al Standardised formal resuscitation training programmes for reducing mortality and morbidity in newborn infants. Cochrane Database Syst Rev 2015:CD009106 (accessed 9 Oct 2016). doi:10.1002/14651858.CD009106.pub2 26337958 10.1002/14651858.CD009106.pub2 PMC9219024

[12] Cochrane Effective Practice and Organisation of Care. Suggested risk of bias criteria for EPOC reviews. http://epoc.cochrane.org/sites/epoc.cochrane.org/files/public/uploads/Resources-for-authors2017/suggested_risk_of_bias_criteria_for_epoc_reviews.pdf (accessed 27 Sep 2017).

[13] Cochrane Training. Cochrane handbook for systematic reviews of interventions. http://training.cochrane.org/handbook (accessed 8 Oct 2016).

[14] The Cochrane Collaboration. Review Manager (RevMan) [Computer program]. Version 5.3. Copenhagen: The Nordic Cochrane Centre, 2014.

[15] GRADEpro | GDT. GRADE’s software for summary of findingstables, health technology assessmentand guidelines. https://gradepro.org/ (accessed 27 Sep 2017).

[16] GRADE. GRADE handbook (SA version). http://gdt.guidelinedevelopment.org/app/handbook/handbook.html (accessed 8 Oct 2016).

[17] Carlo WA, Goudar SS, Jehan I, et al High mortality rates for very low birth weight infants in developing countries despite training. Pediatrics 2010;126:e1072–e1080. doi:10.1542/peds.2010-1183 20937655 10.1542/peds.2010-1183 PMC3918943

[18] Carlo WA, Goudar SS, Jehan I, et al Newborn-care training and perinatal mortality in developing countries. N Engl J Med 2010;362:614–23. doi:10.1056/NEJMsa0806033 20164485 10.1056/NEJMsa0806033 PMC3565382

[19] Opiyo N, Were F, Govedi F, et al Effect of newborn resuscitation training on health worker practices in Pumwani Hospital, Kenya. PLoS One 2008;3:e1599 doi:10.1371/journal.pone.0001599 18270586 10.1371/journal.pone.0001599 PMC2229665

[20] Bang AT, Bang RA, Baitule SB, et al Effect of home-based neonatal care and management of sepsis on neonatal mortality: field trial in rural India. Lancet 1999;354:1955–61. doi:10.1016/S0140-6736(99)03046-9 10622298 10.1016/S0140-6736(99)03046-9

[21] Gill CJ, Phiri-Mazala G, Guerina NG, et al Effect of training traditional birth attendants on neonatal mortality (lufwanyama neonatal survival project): randomised controlled study. BMJ 2011;342:d346 doi:10.1136/bmj.d346 21292711 10.1136/bmj.d346 PMC3032994

[22] Msemo G, Massawe A, Mmbando D, et al Newborn mortality and fresh stillbirth rates in tanzania after helping babies breathe training. Pediatrics 2013;131:e353–e360. doi:10.1542/peds.2012-1795 23339223 10.1542/peds.2012-1795

[23] Goudar SS, Somannavar MS, Clark R, et al Stillbirth and newborn mortality in India after helping babies breathe training. Pediatrics 2013;131:e344–e352. doi:10.1542/peds.2012-2112 23339215 10.1542/peds.2012-2112

[24] Deorari AK, Paul VK, Singh M, et al Impact of education and training on neonatal resuscitation practices in 14 teaching hospitals in India. Ann Trop Paediatr 2001;21:29–33. doi:10.1080/02724930123814 11284243

[25] O’Hare BA, Nakakeeto M, Southall DP A pilot study to determine if nurses trained in basic neonatal resuscitation would impact the outcome of neonates delivered in Kampala, Uganda. J Trop Pediatr 2006;52:376–9. doi:10.1093/tropej/fml027 16782724 10.1093/tropej/fml027

[26] Zhu XY, Fang HQ, Zeng SP, et al The impact of the neonatal resuscitation program guidelines (NRPG) on the neonatal mortality in a hospital in Zhuhai, China. Singapore Med J 1997;38:485–7. 9550910

[27] Bellad RM, Bang A, Carlo WA, et al A pre-post study of a multi-country scale up of resuscitation training of facility birth attendants: does Helping Babies Breathe training save lives? BMC Pregnancy Childbirth 2016;16:222 doi:10.1186/s12884-016-0997-6 27527831 10.1186/s12884-016-0997-6 PMC5477802

[28] Jeffery HE, Kocova M, Tozija F, et al The impact of evidence-based education on a perinatal capacity-building initiative in macedonia. Med Educ 2004;38:435–47. doi:10.1046/j.1365-2923.2004.01785.x 15025645 10.1046/j.1365-2923.2004.01785.x

[29] Sorensen BL, Rasch V, Massawe S, et al Impact of ALSO training on the management of prolonged labor and neonatal care at kagera regional hospital, tanzania. Int J Gynaecol Obstet 2010;111:8–12. doi:10.1016/j.ijgo.2010.04.031 20646704 10.1016/j.ijgo.2010.04.031

[30] Vakrilova L, Elleau C, Slŭncheva B [French-Bulgarian program "Resuscitation of the newborn in a delivery room"--results and perspectives]. Akush Ginekol 2005;44:35–40. 16028390

[31] Boo NY Neonatal resuscitation programme in Malaysia: an eight-year experience. Singapore Med J 2009;50:152–9. 19296030

[32] Hole MK, Olmsted K, Kiromera A, et al A neonatal resuscitation curriculum in Malawi, Africa: did it change in-hospital mortality? Int J Pediatr 2012;2012:1–8. doi:10.1155/2012/408689 10.1155/2012/408689 PMC3228395 22164184

[33] Kc A, Wrammert J, Clark RB, et al Reducing perinatal mortality in nepal using helping babies breathe. Pediatrics 2016;137:e20150117 doi:10.1542/peds.2015-0117 27225317 10.1542/peds.2015-0117

[34] Patel A, Bang A, Kurhe K, et al Impact of implementation of ‘helping babies breathe (HBB)’ training program on all cause and asphyxia specific mortality in selected health facilities. Unpubl Data 2013;16:364.

[35] Bang AT, Bang RA, Tale O, et al Reduction in pneumonia mortality and total childhood mortality by means of community-based intervention trial in Gadchiroli, India. Lancet 1990;336:201–6. doi:10.1016/0140-6736(90)91733-Q 1973770 10.1016/0140-6736(90)91733-q

[36] O’Rourke K, Howard-Grabman L, Seoane G Impact of community organization of women on perinatal outcomes in rural Bolivia. Rev Panam Salud Publica 1998;3:9–14. doi:10.1590/S1020-49891998000100002 9503957 10.1590/s1020-49891998000100002

[37] Pasha O, Goldenberg RL, McClure EM, et al Communities, birth attendants and health facilities: a continuum of emergency maternal and newborn care (the Global Network’s EmONC trial). BMC Pregnancy Childbirth 2010;10:82 doi:10.1186/1471-2393-10-82 21156060 10.1186/1471-2393-10-82 PMC3017016

[38] Pasha O, McClure EM, Wright LL, et al A combined community- and facility-based approach to improve pregnancy outcomes in low-resource settings: a global network cluster randomized trial. BMC Med 2013;11:215 doi:10.1186/1741-7015-11-215 24090370 10.1186/1741-7015-11-215 PMC3853358

[39] Kirkwood BR, Manu A, ten Asbroek AH, et al Effect of the newhints home-visits intervention on neonatal mortality rate and care practices in ghana: a cluster randomised controlled trial. Lancet 2013;381:2184–92. doi:10.1016/S0140-6736(13)60095-1 23578528 10.1016/S0140-6736(13)60095-1

[40] Kumar V, Kumar A, Das V, et al Community-driven impact of a newborn-focused behavioral intervention on maternal health in Shivgarh, India. Int J Gynaecol Obstet 2012;117:48–55. doi:10.1016/j.ijgo.2011.10.031 22281244 10.1016/j.ijgo.2011.10.031

[41] Kumar V, Mohanty S, Kumar A, et al Effect of community-based behaviour change management on neonatal mortality in Shivgarh, Uttar Pradesh, India: a cluster-randomised controlled trial. Lancet 2008;372:1151–62. doi:10.1016/S0140-6736(08)61483-X 18926277 10.1016/S0140-6736(08)61483-X

[42] Bhutta ZA, Soofi S, Cousens S, et al Improvement of perinatal and newborn care in rural Pakistan through community-based strategies: a cluster-randomised effectiveness trial. Lancet 2011;377:403–12. doi:10.1016/S0140-6736(10)62274-X 21239052 10.1016/S0140-6736(10)62274-X

[43] Baqui AH, El-Arifeen S, Darmstadt GL, et al Effect of community-based newborn-care intervention package implemented through two service-delivery strategies in Sylhet district, Bangladesh: a cluster-randomised controlled trial. Lancet 2008;371:1936–44. doi:10.1016/S0140-6736(08)60835-1 18539225 10.1016/S0140-6736(08)60835-1

[44] Tripathy P, Nair N, Barnett S, et al Effect of a participatory intervention with women’s groups on birth outcomes and maternal depression in jharkhand and orissa, india: a cluster-randomised controlled trial. Lancet 2010;375:1182–92. doi:10.1016/S0140-6736(09)62042-0 20207411 10.1016/S0140-6736(09)62042-0

[45] Manandhar DS, Osrin D, Shrestha BP, et al Effect of a participatory intervention with women’s groups on birth outcomes in Nepal: cluster-randomised controlled trial. Lancet 2004;364:970–9. doi:10.1016/S0140-6736(04)17021-9 15364188 10.1016/S0140-6736(04)17021-9

[46] Azad K, Barnett S, Banerjee B, et al Effect of scaling up women’s groups on birth outcomes in three rural districts in bangladesh: a cluster-randomised controlled trial. Lancet 2010;375:1193–202. doi:10.1016/S0140-6736(10)60142-0 20207412 10.1016/S0140-6736(10)60142-0

[47] Pratinidhi A, Shah U, Shrotri A, et al Risk-approach strategy in neonatal care. Bull World Health Organ 1986;64:291–7. 3488845 PMC2490944

[48] Daga SR, Fernandes CJ, Soares M, et al Clinical profile of severe birth asphyxia. Indian Pediatr 1991;28:485–8. 1752675

[49] Chomba E, McClure EM, Wright LL, et al Effect of WHO newborn care training on neonatal mortality by education. Ambul Pediatr 2008;8:300–4. doi:10.1016/j.ambp.2008.04.006 18922503 10.1016/j.ambp.2008.04.006 PMC2592550

[50] Berglund A, Lefevre-Cholay H, Bacci A, et al Successful implementation of evidence-based routines in Ukrainian maternities. Acta Obstet Gynecol Scand 2010;89:230–7. doi:10.3109/00016340903479894 20121338 10.3109/00016340903479894

[51] Mufti P, Setna F, Nazir K Early neonatal mortality: effects of interventions on survival of low birth babies weighing 1000-2000g. J Pak Med Assoc 2006;56:174–6. 16711339

[52] Sen A, Mahalanabis D, Singh AK, et al Impact of a district level sick newborn care unit on neonatal mortality rate: 2-year follow-up. J Perinatol 2009;29:150–5. doi:10.1038/jp.2008.177 18946480 10.1038/jp.2008.177

[53] Patel D, Piotrowski ZH, Nelson MR, et al Effect of a statewide neonatal resuscitation training program on Apgar scores among high-risk neonates in Illinois. Pediatrics 2001;107:648–55. doi:10.1542/peds.107.4.648 11335738 10.1542/peds.107.4.648

[54] Patel D, Piotrowski ZH Positive changes among very low birth weight infant apgar scores that are associated with the neonatal resuscitation program in Illinois. J Perinatol 2002;22:386–90. doi:10.1038/sj.jp.7210751 12082474 10.1038/sj.jp.7210751

[55] Draycott T, Sibanda T, Owen L, et al Does training in obstetric emergencies improve neonatal outcome? BJOG 2006;113:177–82. doi:10.1111/j.1471-0528.2006.00800.x 16411995 10.1111/j.1471-0528.2006.00800.x

[56] Duran R, Görker I, Küçükuğurluoğlu Y, et al Effect of neonatal resuscitation courses on long-term neurodevelopmental outcomes of newborn infants with perinatal asphyxia. Pediatr Int 2012;54:56–9. doi:10.1111/j.1442-200X.2011.03463.x 21895865 10.1111/j.1442-200X.2011.03463.x

[57] Duran R, Aladağ N, Vatansever U, et al Proficiency and knowledge gained and retained by pediatric residents after neonatal resuscitation course. Pediatr Int 2008;50:644–7. doi:10.1111/j.1442-200X.2008.02637.x 19261112 10.1111/j.1442-200X.2008.02637.x

[58] Xu T, Wang H, Gong L, et al The impact of an intervention package promoting effective neonatal resuscitation training in rural China. Resuscitation 2014;85:253–9. doi:10.1016/j.resuscitation.2013.10.020 24176723 10.1016/j.resuscitation.2013.10.020

[59] Bookman L, Engmann C, Srofenyoh E, et al Educational impact of a hospital-based neonatal resuscitation program in Ghana. Resuscitation 2010;81:1180–2. doi:10.1016/j.resuscitation.2010.04.034 20599314 10.1016/j.resuscitation.2010.04.034

[60] Hoban R, Bucher S, Neuman I, et al ’Helping babies breathe' training in sub-saharan africa: educational impact and learner impressions. J Trop Pediatr 2013;59:180–6. doi:10.1093/tropej/fms077 23335632 10.1093/tropej/fms077

[61] Singhal N, Lockyer J, Fidler H, et al Helping babies breathe: global neonatal resuscitation program development and formative educational evaluation. Resuscitation 2012;83:90–6. doi:10.1016/j.resuscitation.2011.07.010 21763669 10.1016/j.resuscitation.2011.07.010

[62] Enweronu-Laryea C, Engmann C, Osafo A, et al Evaluating the effectiveness of a strategy for teaching neonatal resuscitation in west africa. Resuscitation 2009;80:1308–11. doi:10.1016/j.resuscitation.2009.08.005 19720439 10.1016/j.resuscitation.2009.08.005

[63] Ryan CA, Ahmed S, Abdullah H, et al Dissemination and evaluation of AAP/AHA neonatal resuscitation programme in ireland. Ir Med J 1998;91:51–2. 9617029

[64] Halamek LP, Kaegi DM, Gaba DM, et al Time for a new paradigm in pediatric medical education: teaching neonatal resuscitation in a simulated delivery room environment. Pediatrics 2000;106:e45 doi:10.1542/peds.106.4.e45 11015540 10.1542/peds.106.4.e45

[65] Thomas EJ, Williams AL, Reichman EF, et al Team training in the neonatal resuscitation program for interns: teamwork and quality of resuscitations. Pediatrics 2010;125:539–46. doi:10.1542/peds.2009-1635 20156896 10.1542/peds.2009-1635

[66] Thomas EJ, Taggart B, Crandell S, et al Teaching teamwork during the neonatal resuscitation program: a randomized trial. J Perinatol 2007;27:409–14. doi:10.1038/sj.jp.7211771 17538634 10.1038/sj.jp.7211771

[67] Couper ID, Thurley JD, Hugo JF The neonatal resuscitation training project in rural south africa. Rural Remote Health 2005;5:459. 16241856

[68] Nadel FM, Lavelle JM, Fein JA, et al Assessing pediatric senior residents' training in resuscitation: fund of knowledge, technical skills, and perception of confidence. Pediatr Emerg Care 2000;16:73–6. doi:10.1097/00006565-200004000-00001 10784204 10.1097/00006565-200004000-00001

[69] Nadel FM, Lavelle JM, Fein JA, et al Teaching resuscitation to pediatric residents: the effects of an intervention. Arch Pediatr Adolesc Med 2000;154:1049–54. 11030858 10.1001/archpedi.154.10.1049

[70] Kurosawa H, Ikeyama T, Achuff P, et al A randomized, controlled trial of in situ pediatric advanced life support recertification ("pediatric advanced life support reconstructed") compared with standard pediatric advanced life support recertification for ICU frontline providers*. Crit Care Med 2014;42:610–8. doi:10.1097/CCM.0000000000000024 24231759 10.1097/CCM.0000000000000024

[71] Ergenekon E, Koç E, Atalay Y, et al Neonatal resuscitation course experience in turkey. Resuscitation 2000;45:225–7. doi:10.1016/S0300-9572(00)00179-9 10959023 10.1016/s0300-9572(00)00179-9

[72] Quan L, Shugerman RP, Kunkel NC, et al Evaluation of resuscitation skills in new residents before and after pediatric advanced life support course. Pediatrics 2001;108:e110 doi:10.1542/peds.108.6.e110 11731637 10.1542/peds.108.6.e110

[73] Curran V, Fleet L, White S, et al A randomized controlled study of manikin simulator fidelity on neonatal resuscitation program learning outcomes. Adv Health Sci Educ Theory Pract 2015;20:205–18. doi:10.1007/s10459-014-9522-8 24916954 10.1007/s10459-014-9522-8

[74] Ersdal HL, Vossius C, Bayo E, et al A one-day "helping babies breathe" course improves simulated performance but not clinical management of neonates. Resuscitation 2013;84:1422–7. doi:10.1016/j.resuscitation.2013.04.005 23612024 10.1016/j.resuscitation.2013.04.005

[75] Ersdal HL, Singhal N Resuscitation in resource-limited settings. Semin Fetal Neonatal Med 2013;18:373–8. doi:10.1016/j.siny.2013.07.001 23896083 10.1016/j.siny.2013.07.001

[76] Matendo R, Engmann C, Ditekemena J, et al Reduced perinatal mortality following enhanced training of birth attendants in the democratic republic of congo: a time-dependent effect. BMC Med 2011;9:93 doi:10.1186/1741-7015-9-93 21816050 10.1186/1741-7015-9-93 PMC3171324

[77] Vossius C, Lotto E, Lyanga S, et al Cost-effectiveness of the "helping babies breathe" program in a missionary hospital in rural Tanzania. PLoS One 2014;9:e102080 doi:10.1371/journal.pone.0102080 25006802 10.1371/journal.pone.0102080 PMC4090230

[78] Edmond KM, Quigley MA, Zandoh C, et al Aetiology of stillbirths and neonatal deaths in rural Ghana: implications for health programming in developing countries. Paediatr Perinat Epidemiol 2008;22:430–7. doi:10.1111/j.1365-3016.2008.00961.x 18782251 10.1111/j.1365-3016.2008.00961.x

[79] Darmstadt GL, Bhutta ZA, Cousens S, et al Evidence-based, cost-effective interventions: how many newborn babies can we save? Lancet 2005;365:977–88. doi:10.1016/S0140-6736(05)71088-6 15767001 10.1016/S0140-6736(05)71088-6

[80] Bhutta ZA, Darmstadt GL, Hasan BS, et al Community-based interventions for improving perinatal and neonatal health outcomes in developing countries: a review of the evidence. Pediatrics 2005;115:519–617. doi:10.1542/peds.2004-1441 15866863 10.1542/peds.2004-1441

[81] Kumbani L, Bjune G, Chirwa E, et al Why some women fail to give birth at health facilities: a qualitative study of women’s perceptions of perinatal care from rural southern malawi. Reprod Health 2013;10:9 doi:10.1186/1742-4755-10-9 23394229 10.1186/1742-4755-10-9 PMC3585850

[82] Yakoob MY, Menezes EV, Soomro T, et al Reducing stillbirths: behavioural and nutritional interventions before and during pregnancy. BMC Pregnancy Childbirth 2009;9:S3 doi:10.1186/1471-2393-9-S1-S3 19426466 10.1186/1471-2393-9-S1-S3 PMC2679409

[83] Bang A, Bellad R, Gisore P, et al Implementation and evaluation of the helping babies breathe curriculum in three resource limited settings: does helping babies breathe save lives? a study protocol. BMC Pregnancy Childbirth 2014;14:116 doi:10.1186/1471-2393-14-116 24670013 10.1186/1471-2393-14-116 PMC4021423

[84] Bang A, Patel A, Bellad R, et al Helping Babies Breathe (HBB) training: What happens to knowledge and skills over time? BMC Pregnancy Childbirth 2016;16:364 doi:10.1186/s12884-016-1141-3 27875999 10.1186/s12884-016-1141-3 PMC5120476

